# Supplementary material for: Quality of Life Following Pelvic Organ Prolapse Treatments in Women: A Systematic Review and Meta-Analysis
Source: J Clin Med. 2022 Dec 1;11(23):7166. doi: 10.3390/jcm11237166 (PMC9738239; doi:10.3390/jcm11237166)
Supplement: Supplementary file 1 [file jcm-11-07166-s001.zip › jcm-2003453-supplementary.pdf]

# Supplementary Material

## Quality of life following pelvic organ prolapse treatments in women: A systematic review and meta-analysis

**Search query**

## PubMed Search Query:

1-“ Pelvic Organ Prolapse”[mh] Pelvic Organ Prolapse[tiab] OR Pelvic Organ Prolapses [tiab] OR Urogenital Prolapse[tiab] OR Urogenital Prolapses[tiab] OR Vaginal Vault Prolapse[tiab] OR Vaginal Vault Prolapses[tiab] OR Cystocele[tiab] OR Rectal Prolapse[tiab] OR Uterine Prolapse[tiab] OR Visceral Prolapse[tiab]

2-“ Quality of Life”[mh] OR Quality of Life [tiab]

3- Surgery[tiab] OR Pessaries[tiab] OR pessary[tiab]

4- ((" Pelvic Organ Prolapse"[mh] Pelvic Organ Prolapse[tiab] OR Pelvic Organ Prolapses [tiab] OR Urogenital Prolapse[tiab] OR Urogenital Prolapses[tiab] OR Vaginal Vault Prolapse[tiab] OR Vaginal Vault Prolapses[tiab] OR Cystocele[tiab] OR Rectal Prolapse[tiab] OR Uterine Prolapse[tiab] OR Visceral Prolapse[tiab]) AND (" Quality of Life"[mh] OR Quality of Life [tiab])) AND (Surgery[tiab] OR Pessaries[tiab] OR pessary[tiab])

## Scopus

1- “Pelvic Organ Prolapse” OR “ Pelvic Organ Prolapses “ OR “ Urogenital Prolapse” OR “ Urogenital Prolapses” OR “ Vaginal Vault Prolapse” OR “ Vaginal Vault Prolapses” OR “ Cystocele” OR “ Rectal Prolapse” OR “ Uterine Prolapse” OR “ Visceral Prolapse”

2- “ Quality of life”

3- “ Surgery” OR “ Pessaries” OR “ pessary”

( TITLE-ABS-KEY ( "Pelvic Organ Prolapse"  OR  " Pelvic Organ Prolapses "  OR  " Urogenital Prolapse"  OR  " Urogenital Prolapses"  OR  " Vaginal Vault Prolapse"  OR  " Vaginal Vault Prolapses"  OR  " Cystocele"  OR  " Rectal Prolapse"  OR  " Uterine Prolapse"  OR  " Visceral

Prolapse" ) ) AND ( TITLE-ABS-KEY ( " Quality of life" ) ) AND ( TITLE-ABS-KEY ( " Surgery" OR " Pessaries" OR " pessary" ) )

## Web of Science

1- “Pelvic Organ Prolapse” OR “ Pelvic Organ Prolapses “ OR “ Urogenital Prolapse” OR “ Urogenital Prolapses” OR “ Vaginal Vault Prolapse” OR “ Vaginal Vault Prolapses” OR “ Cystocele” OR “ Rectal Prolapse” OR “ Uterine Prolapse” OR “ Visceral Prolapse”

2- “ Quality of life”

3- “ Surgery” OR “ Pessaries” OR “ pessary”

TS=(“Pelvic Organ Prolapse” OR “ Pelvic Organ Prolapses “ OR “ Urogenital Prolapse” OR “ Urogenital Prolapses” OR “ Vaginal Vault Prolapse” OR “ Vaginal Vault Prolapses” OR “ Cystocele” OR “ Rectal Prolapse” OR “ Uterine Prolapse” OR “ Visceral Prolapse” ) AND TS=(“ Quality of life” ) AND TS=(“ Surgery” OR “ Pessaries” OR “ pessary”)
